# Editorial Comment: The significance of intraoperative renal pelvic urine and stone cultures for patients at a high risk of post-ureteroscopy systemic inflammatory response syndrome

**DOI:** 10.1590/S1677-5538.IBJU.2020.05.06

**Published:** 2020-07-31

**Authors:** Alexandre Danilovic

**Affiliations:** 1 Hospital das Clínicas da Faculdade de Medicina da USP Serviço de Urologia São Paulo SP Brasil Serviço de Urologia, Hospital das Clínicas da Faculdade de Medicina da USP - HCFMUSP, São Paulo, SP, Brasil

Yoshida S ^1^, Takazawa R ^2^, Uchida Y ^1^, Kohno Y ^1^, Waseda Y ^1^, Tsujii T ^1^

## COMMENT

Infectious complications are among the most feared complications of retrograde intrarenal surgery (RIRS). Untreated positive pre-operative bladder urine culture (PBUC) is a contraindication for RIRS, but infectious complications may occur even with prophylactic antibiotics and negative PBUC (1). The role of renal pelvic urine culture (RPUC) and stone culture (SC) are well known in patients undergoing percutaneous nephrolithotomy (2). However, its role in RIRS is not established.

This prospective study investigated the associations among the results of PBUC, RPUC, and SC. PBUC was positive in 49.6% of the patients. Even after adequate antibiotic administration, RPUC was positive in 19.2% and SC was positive in 15.2% of the patients. Moreover, bacterial species detected by PBUC were not necessarily consistent with those of RPUC and/or SC. Systemic inflammatory response syndrome (SIRS) occurred in 10.3% of the patients. Female gender (OR=2.81), struvite calculi (OR=4.95) and positive RPUC (OR=3.83) were significant risk factors of SIRS after RIRS.

Therefore, obtaining a RPUC during RIRS is highly recommended because it can be useful for selecting appropriate antibiotics.
